# Potential distribution prediction of *Amaranthus palmeri* S. Watson in China under current and future climate scenarios

**DOI:** 10.1002/ece3.9505

**Published:** 2022-12-12

**Authors:** Xinyi Zhang, Jian Zhao, Miaomiao Wang, Zhipeng Li, Sheng Lin, Hong Chen

**Affiliations:** ^1^ Institute of Digital Agriculture Fujian Academy of Agricultural Sciences Fuzhou China; ^2^ State Key Laboratory for Ecological Pest Control of Fujian and Taiwan Crops Institute of Applied Ecology, Fujian Agriculture and Forestry University Fuzhou China

**Keywords:** *Amaranthus palmeri* S. Watson, climate change, MaxEnt model, potential distribution, suitability

## Abstract

The vicious invasive alien plant *Amaranthus palmeri* poses a serious threat to ecological security and food security due to its strong adaptability, competitiveness, and herbicide resistance. Predicting its potential habitats under current and future climate change is critical for monitoring and early warning. In this study, we used two sets of climate data, namely, WorldClim1.4 and RCPs (the historical climate data of WorldClim version 1.4 and future climate data of RCPs), WorldClim2.1 and SSPs (the historical climate data of WorldClim version 2.1 and future climate data of SSPs), to analyze the dominant environmental variables affecting the habitat suitability and predict the potential distribution of *A. palmeri* to climate change in China based on the MaxEnt model. The results show that (i) Temperature has a greater impact on the distribution of *A. palmeri*. The relative contributions of temperature‐related variables count to 70% or more, and the annual mean temperature (bio1) reached more than 40%. (ii) At present, the potentially suitable area is widely distributed in the central‐east and parts of southwest China, and the high suitable area is focused on the North China Plain. The potential suitable area predicted by WorldClim1.4 and WorldClim2.1 both accounts for about 31% of China's total land area. (iii) Future climate change will expand the suitable habitats to high latitudes and altitudes. The overall suitable area maximum increased to 44.93% under SSPs and 38.91% under RCPs. We conclude that climate change would increase the risk of *A. palmeri* expanding to high latitudes and altitudes, the results have practical implications for the effective long‐term management in response to the global warming of *A. palmeri*.

## Graphical abstract

*Amaranthus palmeri* S. Watson, a malignant invasive weed, its invasion potential has not been fully exploited in China. In the future, it will expand significantly to higher latitudes and altitudes in response to global warming.

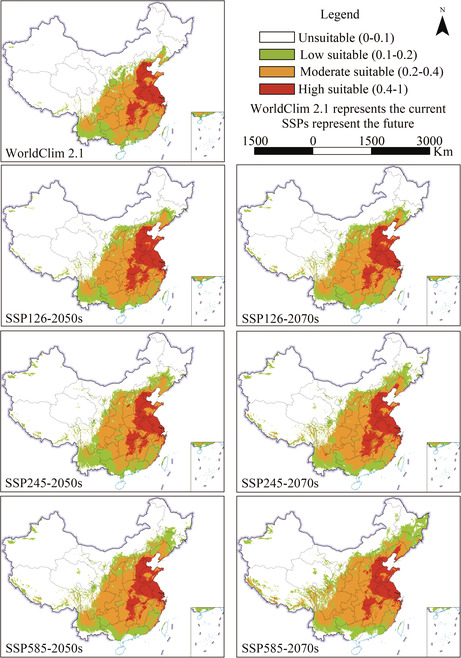

## 1 INTRODUCTION

Biosecurity, biodiversity and biological invasion are interactive and closely linked. Biosecurity is related to ecological civilization, and a good ecological environment is an important foundation for agriculture's high quality and green development. Protecting biodiversity and preventing the invasion of alien species are essential parts of biosecurity, and biological invasion is one of the direct causes of biodiversity decline (McGeoch et al., 2010). Invasive alien species threaten global biodiversity and cause immeasurable damage to ecology by altering the structure and function of ecosystems and disrupting key biological interactions (Mitchell et al., 2006). In addition, the total minimum economic cost of the global biological invasion reached US$1.288 trillion (2017 US dollars) between 1970 and 2017 (Diagne et al., 2021). Nowadays, with the accelerated process of world globalization, the spread risk of invasive alien species is increasing (Seebens et al., 2017, 2018). And the external biological threats and internal biological risks coexist reciprocally. Therefore, it is essential to strengthen the prevention and control of invasive alien species.

*Amaranthus palmeri* S. Watson is an annual C_4_ herb originating from the southwestern United States and northwestern Mexico (Sauer, 1957). In China, *A. palmeri* was first discovered in Beijing in 1985 (Li, 2003). Now, it is mainly distributed in Beijing, Tianjin, Henan, and so on (Li, Cao, et al., 2021; Lv et al., 2015; Mo et al., 2017). It threatens biodiversity and causes a significant decline in crop production by competing for light, water, nutrition, and living space with native plants, existing as a host for various nematode species, and allelopathic effect (Kaspary et al., 2021; Menges, 1987; Ward et al., 2013). According to the statistics of the United States from 2001 to 2010, the most serious yield losses of sweet potato, corn, soybean, peanut, cotton and sorghum caused by *A. palmeri* were 94%, 91%, 79%, 68%, 59%, and 50%, respectively (Ward et al., 2013). Furthermore, according to a study in Tianjin, China, the relative dominance of *A. palmeri* was negatively correlated with the Shannon‐Wiener diversity index and evenness. With the increase of the invasion effect of *A. palmeri*, the species richness decreased, which seriously affected the biodiversity (Li, Mo, et al., 2021).

At present, the control of *A. palmeri* mainly relies on applying herbicides. However, multiple populations of *A. palmeri* resistant to herbicides have developed worldwide due to the cultivation of herbicide‐resistant crops and the long‐term use of herbicides (Heap, 2022; Ji et al., 2020). Furthermore, as a dioecious plant, *A. palmeri* can transfer herbicide‐resistance genes to other closely related weeds through interspecific hybridization, posing the risk of forming a “super weed” (Molin et al., 2016; Ward et al., 2013). China is a big agricultural country with limited arable land. Once *A. palmeri* is established in a certain place, it is difficult to be completely eradicated it, which will inevitably affect China's agricultural production (Xu et al., 2013). Based on the principle that prevention is superior to governance, it is urgent to assess the risk of *A. palmeri* to identify the dominant environmental variables that affect the suitable habitat and judge the suitable degree of *A. palmeri* in China, which contributes to early warning and rapid response.

Climate is important in determining species distribution (Pyšek et al., 2020). Temperature and rainfall indirectly affect the interaction between native and exotic species by influencing species' phenological and physiological activities, thereby affecting the spatial pattern of species (Robinson et al., 2020). Global climate change and extreme climate events could completely alter species' spatial distribution and prevalence (Manzoor et al., 2021; Marchioro & Krechemer, 2021). In addition, rising temperatures and changes in the timing and frequency of rainfall may exacerbate the negative effects of *A. palmeri* on crops (Davis et al., 2015; McDonald et al., 2009). Therefore, exploring the spatial pattern of suitable areas for *A. palmeri* under future climate change is important for prospective risk warning.

Species Distribution Models (SDMs) correlate species' distribution data with environmental variables and study the potential spatial pattern of species according to the specific algorithm (Phillips et al., 2006; Yates et al., 2018). The results can be interpreted as the probability of species occurrence, habitat suitability, or species richness (Tu et al., 2021; Zhu et al., 2013). Over the past few decades, many studies applied SDMs to risk assessment of invasive alien species and analysis of the impact of climate change on species distribution (Adhikari et al., 2021; Hong et al., 2021). According to the statistic, the Maximum Entropy (MaxEnt) model ranked first among 17 modeling algorithms used in articles on SDMs and invasive alien plants from 1996 to 2019 (Silva et al., 2021). Many researchers have proved that the MaxEnt model performs well in prediction accuracy and operation (Hong et al., 2021; Phillips & Dudík, 2008; Roger et al., 2015) and has become one of the most popular species distribution modeling tools (Merow et al., 2013).

At present, the study of *A. palmeri* mainly focuses on the resistance and biological and physiological mechanisms (Ji et al., 2022; Maxwel et al., 2020; Mesgaran et al., 2021; Meyer et al., 2020). In addition, there are studies on predicting suitable habitats for *A. palmeri*. However, most of the studies are focused on the potential distribution predictions under the current climatic conditions, lacking future projection. And there is no detailed explanation for the dominant environmental variables that affect growth suitability. For example, Xu et al. (2013), Li et al. (2015) and Zhao (2018) used CLIMEX, MaxEnt, and BIOCLIM models to predict the potential distribution of *A. palmeri* in China under current climatic conditions, respectively. According to another study, CLIMEX was used to predict the potential geographical distribution of *A. palmeri* under current and future climate scenarios. However, only the Representative Concentration Pathway (RCP) 8.5 emission scenario of the 2050s was adopted, and there was no detailed description of the suitability of *A. palmeri* in China (Kistner & Hatfield, 2018). The most critical theoretical basis for applying the MaxEnt model is the conservation of species niches (Zhu et al., 2013). Li et al. (2015) pointed out that the niche of *A. palmeri* was conservative in the process of invasion, which laid a foundation for the application of the MaxEnt model.

*Amaranthus palmeri* may exhibit a competitive disadvantage in high latitudes and altitudes (Ward et al., 2013). Will it expand northwards in response to climate change in the future? If it expands, how is the expansion? All need further research.

This study used the MaxEnt model to investigate the dominant environmental variables and predict the potentially suitable areas of *A. palmeri* in China under current and future climate change. The specific objectives are (i) to identify the dominant environmental variables affecting the suitable habitat of *A. palmeri* and (ii) to predict potential distribution and migration in China under current and future climate scenarios. The outcome of this study may provide a scientific basis with practical significance for the long‐term management of *A. palmeri*, and it is of great importance for protecting ecological security, biodiversity, and food security.

## 2 MATERIALS AND METHODS

### 2.1 Occurrence data

**FIGURE 1:**
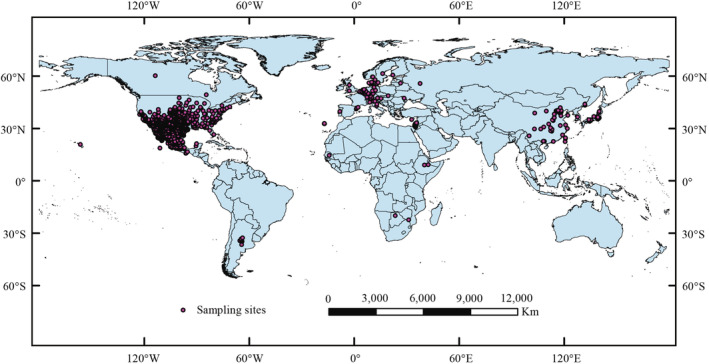
Spatial distribution of the sampling records of *Amaranthus palmeri*.

The spatial distribution data of *A. palmeri* was downloaded from Global Biodiversity Information Facility (GBIF; https://www.gbif.org/), Southwest Environmental Information Network (SEINet; https://swbiodiversity.org/seinet/index.php), Center for Agriculture and Bioscience International (CABI; https://www.plantwise.org/knowledgebank/), Plant Photo Bank of China (PPBC; http://ppbc.iplant.cn/), Chinese Virtual Herbarium (CVH; https://www.cvh.ac.cn/), National Specimen Information Infrastructure (NSII; http://www.nsii.org.cn/), and some records from literatures (Cao et al., 2020; Li, 2003; Li et al., 2015, 2017; Mo et al., 2017; Wu et al., 2018; Zhang, 2020). We kept the records with accurate latitude and longitude from the above data. In addition, records with detailed geographic locations were obtained from CVH, NSII, PPBC, and literatures. Map World (https://www.tianditu.gov.cn) was used to determine geographical coordinates. After deleting the duplicate and inaccuracy records, 2141 samples were retained. To reduce sampling bias, the create fishnet function of ArcGIS software version 10.2 was used to generate the fishnet regarding the spatial resolutions (2.5 min) of the environment layer. It was retained when there was only one occurrence of data in each geographic space. Their average value was calculated and retained when there were more than or equal to two distributed data. After filtering, we obtained 1679 effective samples (Figure 1).

### 2.2 Environment variables

The distribution of species at a large geographical spatial scale is mainly related to climate (Hortal et al., 2010; Pearson & Dawson, 2003; Soberón, 2007, 2010). Therefore, our study mainly considers the influence of climate on the distribution of *A. palmeri*. The climate data were downloaded from the WorldClim data website (https://www.worldclim.org/data/index.html) including 19 bioclimatic variables (bio1–bio19). Bioclimatic variables are derived from monthly temperature and rainfall values, which are of more biological significance and are widely used in species distribution modeling and related ecological modeling techniques. In addition, altitude may affect the occurrence by affecting temperature (Azrag et al., 2018). Thus, we added altitude as a terrain factor. The elevation above sea level data was derived from the SRTM elevation data. The description of the environment variables is shown in Table 1. Generally, the higher the spatial resolution of the environmental layer is, the more accurate the model running result is. However, the pressure of the model running will greatly increase. Therefore, we chose the 2.5 min spatial resolution of all environmental layers to ensure high accuracy and good running ability.

**TABLE 1:** Abbreviation and description of environmental variables

Abbreviation	Description	Unit	Abbreviation	Description	Unit
**bio1**	**Annual mean temperature**	**°C**	bio11	Mean temperature of coldest quarter	°C
**bio2**	**Mean diurnal range (mean of monthly [max temp − min temp])**	**°C**	**bio12**	**Annual precipitation**	**mm**
bio3	Isothermality (bio2/bio7) (×100)	/	**bio13**	**Precipitation of wettest month**	**mm**
**bio4**	**Temperature seasonality**	**/**	bio14	Precipitation of driest month	mm
**bio5**	**Max temperature of warmest month**	**°C**	**bio15**	**Precipitation seasonality (coefficient of variation)**	**/**
bio6	Min temperature of coldest month	°C	bio16	Precipitation of wettest quarter	mm
bio7	Temperature annual range (bio5–bio6)	°C	**bio17**	**Precipitation of driest quarter**	**mm**
**bio8**	**Mean temperature of wettest quarter**	**°C**	bio18	Precipitation of warmest quarter	mm
**bio9**	**Mean temperature of driest quarter**	**°C**	**bio19**	**Precipitation of coldest quarter**	**mm**
bio10	Mean temperature of warmest quarter	°C	**elev**	**Elevation above sea level**	**m**

*Note*: The variables being used in the model are marked bold.

The historical climate data include two versions, WorldClim 1.4 for 1960–1990 and WorldClim 2.1 for 1970–2000. There are two versions for future climate scenarios, RCPs (Representative Concentration Pathways) and SSPs (Shared Socio‐economic Pathways). RCPs were calibrated with WorldClim 1.4 as the baseline and proposed in IPCC‐CMIP5. RCPs contain four pathways (RCPs: 2.6, 4.5, 6.0, and 8.5). SSPs were calibrated with WorldClim 2.1 as the baseline and proposed in IPCC‐CMIP6. The RCPs have new versions in CMIP6. These updated scenarios are called SSP1‐2.6, SSP2‐4.5, SSP4‐6.0, and SSP5‐8.5 (SSPs: 126, 245, 460, and 585). At present, most studies have been based on RCPs. However, the SSPs have considered social and economic development and tended to show notably higher climate sensitivity than RCPs. Therefore, we chose two sets of historical climate data and future climate scenarios to conduct a comprehensive analysis.

As for the Global Climate Models (GCMs), we used the BCC‐CSM1‐1 and the BCC‐CSM2‐MR, which are reliable for simulating precipitation and temperature in China. The Beijing Climate Center Climate System Model (BCC‐CSM) is a fully coupled global climate system consisting of atmosphere, land surface, ocean, and sea ice, which is developed on the basis of the Community Climate System Model (CCSM) by improving the process parameterization schemes of the atmosphere and land surface component models (Shi et al., 2020; Wu et al., 2013, 2019). The BCC‐CSM1‐1 predicts that the rise of mean temperature in China by the end of the 21st century is consistent with the CMIP5 multi‐model ensemble mean, and performs well in simulating the historical evolution of global and Chinese mean surface air temperature (Wu et al., 2013, 2014). BCC‐CSM2‐MR improves the spatial pattern of surface air temperature, precipitation, general atmospheric circulation, and other main climate variables at a global scale and regional scale in East Asia due to the new cloud fraction scheme and the indirect influence of aerosols on cloud and precipitation (Wu et al., 2019). The GCMs developed by BCC‐CSM have been used in many studies to simulate the distribution of native and invasive species in China (Ma et al., 2021; Tu et al., 2021; Zhao, Deng, et al., 2021).

Eventually, we selected two sets of current and future climate scenarios, including WorldClim1.4, RCP2.6‐2050s, RCP2.6‐2070s, RCP4.5‐2050s, RCP4.5‐2070s, RCP8.5‐2050s, and RCP8.5‐2070s; WorldClim2.1, SSP126‐2050s, SSP126‐2070s, SSP245‐2050s, SSP245‐2070s, SSP585‐2050s, and SSP585‐2070s.

To avoid the over‐fitting caused by the high correlation of environmental variables, we used the Pearson correlation of IBM SPSS Statistics Software version 21 to test the multicollinearity of bioclimatic variables. Only one meaningful variable was retained when the correlation coefficient |*R*| ≥ .8. The two versions of bioclimatic variables showed similar correlations (Tables 2 and 3). For temperature‐related variables, according to the information that the annual mean temperature plays an important role in the distribution prediction models of *A. palmeri* (Li et al., 2015; Runquist et al., 2019) and the seeds' germination ability of *A. palmeri* is significantly affected by temperature seasonality (Jha et al., 2010), we retained annual mean temperature (bio1) and temperature seasonality (bio4) and removed variables that are highly correlated with them. For precipitation‐related variables, according to the information that the appearance of *A. palmeri* is often related to arid environment, it can quickly respond to the available moisture in the environment (Ehleringer, 1983; Piskackova et al., 2021), and severe flooding conditions may affect its occurrence (Franca et al., 2020), we retained extreme precipitation variable precipitation of wettest month (bio13) and quarterly precipitation variable precipitation of driest quarter (bio17) and removed variables that are highly correlated with them. We retained the remaining variables and finally selected 11 bioclimatic variables and elevation to participate in modeling (Table 1).

**TABLE 2:** The Pearson correlation coefficient of bioclimatic variables for WorldClim 1.4

	bio1	bio2	bio3	bio4	bio5	bio6	bio7	bio8	bio9	bio10	bio11	bio12	bio13	bio14	bio15	bio16	bio17	bio18	bio19
bio1	1																		
bio2	0.110	1																	
bio3	0.571	0.511	1																
bio4	−0.574	0.029	**−0.814**	1															
bio5	0.686	0.545	0.253	0.071	1														
bio6	**0.888**	−0.139	0.631	**−0.830**	0.323	1													
bio7	−0.477	0.476	−0.484	**0.884**	0.287	**−0.813**	1												
bio8	0.484	0.278	0.177	0.038	0.555	0.204	0.135	1											
bio9	0.751	0.227	0.640	−0.629	0.498	0.786	−0.489	0.083	1										
bio10	**0.817**	0.186	0.144	−0.002	**0.905**	0.504	0.046	0.611	0.499	1									
bio11	**0.937**	0.085	0.749	**−0.820**	0.461	**0.969**	−0.698	0.316	**0.806**	0.573	1								
bio12	−0.131	−0.559	−0.374	0.060	−0.334	−0.047	−0.158	−0.107	−0.286	−0.154	−0.142	1							
bio13	0.095	−0.407	−0.034	−0.170	−0.232	0.158	−0.302	0.070	−0.111	−0.050	0.109	0.758	1						
bio14	−0.279	−0.486	−0.562	0.285	−0.296	−0.234	0.055	−0.173	−0.362	−0.161	−0.332	0.777	0.244	1					
bio15	0.370	0.182	0.555	−0.427	0.111	0.380	−0.316	0.170	0.341	0.141	0.434	−0.365	0.266	−0.748	1				
bio16	0.066	−0.419	−0.025	−0.198	−0.277	0.156	−0.328	0.012	−0.110	−0.102	0.101	0.799	**0.983**	0.285	0.215	1			
bio17	−0.263	−0.473	−0.554	0.281	−0.273	−0.225	0.060	−0.161	−0.343	−0.144	−0.319	0.783	0.253	**0.994**	−0.756	0.290	1		
bio18	−0.082	−0.293	−0.174	0.068	−0.237	−0.094	−0.051	0.273	−0.367	−0.092	−0.111	0.724	**0.804**	0.375	0.014	**0.801**	0.379	1	
bio19	−0.071	−0.468	−0.247	−0.071	−0.266	0.120	−0.285	−0.528	0.185	−0.143	−0.022	0.514	0.246	0.520	−0.261	0.291	0.521	−0.074	1

*Note*: The description of environmental variables is shown in Table 1 and the variables with correlation coefficient ≥0.8 are marked in bold.

**TABLE 3:** The Pearson correlation coefficient of bioclimatic variables for WorldClim 2.1

	bio1	bio2	bio3	bio4	bio5	bio6	bio7	bio8	bio9	bio10	bio11	bio12	bio13	bio14	bio15	bio16	bio17	bio18	bio19
bio1	1																		
bio2	0.156	1																	
bio3	0.562	0.553	1																
bio4	−0.549	0.004	**−0.800**	1															
bio5	0.691	0.579	0.271	0.082	1														
bio6	**0.878**	−0.112	0.609	**−0.818**	0.317	1													
bio7	−0.434	0.482	−0.434	**0.869**	0.324	−0.795	1												
bio8	0.484	0.329	0.213	0.036	0.556	0.191	0.165	1											
bio9	0.753	0.220	0.602	−0.604	0.499	0.788	−0.467	0.072	1										
bio10	**0.816**	0.214	0.133	0.031	**0.900**	0.489	0.088	0.596	0.503	1									
bio11	**0.933**	0.126	0.741	**−0.809**	0.463	**0.965**	−0.666	0.319	**0.802**	0.562	1								
bio12	−0.118	−0.557	−0.348	0.027	−0.350	−0.021	−0.203	−0.098	−0.263	−0.155	−0.120	1							
bio13	0.116	−0.388	0.012	−0.223	−0.258	0.190	−0.354	0.087	−0.093	−0.058	0.147	0.753	1						
bio14	−0.269	−0.486	−0.545	0.269	−0.290	−0.215	0.029	−0.173	−0.335	−0.150	−0.319	0.770	0.231	1					
bio15	0.361	0.213	0.565	−0.440	0.092	0.366	−0.306	0.169	0.327	0.115	0.434	−0.366	0.272	−0.741	1				
bio16	0.090	−0.399	0.021	−0.251	−0.295	0.190	−0.379	0.025	−0.085	−0.107	0.142	0.792	**0.982**	0.269	0.223	1			
bio17	−0.264	−0.475	−0.540	0.268	−0.279	−0.213	0.034	−0.167	−0.324	−0.146	−0.314	0.779	0.238	**0.994**	−0.753	0.274	1		
bio18	−0.032	−0.257	−0.101	0.002	−0.221	−0.041	−0.100	0.300	−0.328	−0.072	−0.046	0.718	**0.814**	0.344	0.038	**0.810**	0.351	1	
bio19	−0.082	−0.489	−0.271	−0.066	−0.287	0.124	−0.307	−0.542	0.206	−0.148	−0.032	0.495	0.215	0.516	−0.264	0.267	0.516	−0.102	1

*Note*: The description of environmental variables is shown in Table 1 and the variables with correlation coefficient ≥0.8 are marked in bold.

### 2.3 Model setting and evaluation

In this study, we used the MaxEnt model (version 3.4.1) to predict the potential spatial distribution of *A. palmeri*. The models were set to 10 replicates using the subsample sampling method; 75% of data were randomly selected as the training data sets and the remaining 25% as the test sets. In addition, the random seed was applied for each run to make a different random test or train partition and use a different random subset of the background. The jackknife was used to measure the importance of environmental variables, and the response curve was drawn to analyze the response of the distribution probability of *A. palmeri* to environmental factors. Besides, the output format was adjusted to logistic to better understand the probabilistic relationship between distribution and environmental variables. Other settings remain default. Eventually, we adopted the average result of 10 repeated runs.

The commonly used model evaluation indexes include Kappa (Cohen, 1960), TSS (Allouche et al., 2006), and AUC (Hanley & McNeil, 1982) statistics value. AUC measures the commission and omission error equally, which may be misleading in model evaluation (Lobo et al., 2008). Kappa is affected by species distribution rate (Lantz & Nebenzahl, 1996). TSS equally weights sensitivity and specificity, but different weights may be required for practical application (Allouche et al., 2006). Therefore, we used the test set to calculate AUC, Kappa and TSS to evaluate the model's performance. The range of AUC was [0,1] (Swets, 1988), and the range of Kappa and TSS was [−1,1] (Allouche et al., 2006). In practical application, when AUC > 0.5, Kappa > 0, or TSS > 0, the prediction effect of the model is better than that of random prediction and the judgment of the model is meaningful (Allouche et al., 2006; Swets, 1988). The larger the AUC, Kappa, and TSS values, the higher the accuracy of prediction and the better the consistency.

### 2.4 Division of suitable areas

To quantify the potential suitable areas of *A. palmeri* in China, the clip function of data management in ArcGIS was used to cut the Chinese result from the predicted potential distribution map, and the reclass function was used to classify the suitable areas. Referring to the division method of Zhao, Cui, et al. (2021) and Zhao, Deng, et al. (2021), we used the natural breaks (Jenks) to divide the prediction results. According to the results of Jenks, we reclassified the potential distribution prediction results of *A. palmeri* into four categories, unsuitable (0–0.1), low suitable (0.1–0.2), moderate suitable (0.2–0.4), and high suitable (0.4–1).

For further quantitatively comparing the spatial pattern changes in potential suitable habitats (Jian et al., 2022; Ran et al., 2019), we superimposed distribution layers from the current to the 2050s (current‐2050s) and from the 2050s to the 2070s (2050s–2070s). We took 1, 2, 3, and 4 as the codes of the unsuitable, low, moderate, and high suitable areas, respectively. The reclassification results were superimposed and classified using the ArcGIS's raster calculator. The calculation formula is as follows:

(1) $$\text{Layer}={\text{Layer}}_{1}\times 10+{\text{Layer}}_{2}$$

Layer represents the code for the overlay layer, ${\text{Layer}}_{1}$ represents the code for the first layer, ${\text{Layer}}_{2}$ represents the code for the second layer. The description of the overlay code is shown in Table 4. The first and second digits of the overlay code can be considered suitable levels for the first and second layers of the overlay layer.

**TABLE 4:** The description of the overlay code

Overlay code	Description
11	Unsuitable
22, 33, 44	Suitable level remained the same
12, 13, 14	New (unsuitable to suitable)
23, 24, 34	Suitable level raised
21, 31, 41	Disappeared (suitable to unsuitable)
32, 42, 43	Suitable level dropped

## 3 RESULTS

### 3.1 Model accuracy evaluation

As shown in Table 5, the average AUC values of models running on the two sets of environment data were >0.9, and the average Kappa and TSS values were >0.8. That means the two sets of environmental data prediction models have great accuracy and consistency. The standard deviations of Kappa and TSS of WorldClim2.1 were slightly lower than WorldClim1.4, indicating that the model predicted by WorldClim2.1 might have better stability.

**TABLE 5:** The values of AUC, Kappa, and TSS

Category	WorldClim 1.4	WorldClim 2.1
AUC		
Average	0.9641	0.9729
SD	0.0042	0.0042
Kappa		
Average	0.8090	0.8392
SD	0.0140	0.0119
TSS		
Average	0.8118	0.8393
SD	0.0128	0.0124

### 3.2 Dominant environmental variables

For the dominant environmental variables affecting the suitable habitat of *A. palmeri*, we comprehensively consider the relative contributions of the environmental variables to the models and the results of the jackknife test of variable importance.

Table 6 gives two estimates of relative contributions, including percent contribution and permutation importance. The two estimates of the relative contributions are different because of different calculation methods. Generally, the percent contribution is preferred. According to the results, the percent contribution of annual mean temperature (bio1), mean diurnal range (bio2), temperature seasonality (bio4), and precipitation of coldest quarter (bio19) to the models are all >10%. Their cumulative contribution to WorldClim 1.4 and WorldClim 2.1 is 83.9% and 83.7%, respectively. Overall, the percent contribution of temperature‐related environmental variables is higher than rainfall‐related variables, although the percent contribution in WorldClim 1.4 and WorldClim 2.1 is different.

**TABLE 6:** The estimates of relative contributions of the environmental variables

Variable	WorldClim 1.4		WorldClim 2.1	
	Percent contribution (%)	Permutation importance (%)	Percent contribution (%)	Permutation importance (%)
bio1	41.8	40.5	48.3	57.5
bio2	14.1	6.6	14.6	5.7
bio4	14.7	8.8	10.6	4.4
bio5	0.4	1.0	1.2	0.5
bio8	2.5	5.1	0.9	7.8
bio9	2.8	3.5	5.7	1.5
bio12	1.4	5.5	1.4	5.1
bio13	4.3	0.1	2.8	0.0
bio15	0.5	3.9	0.3	2.6
bio17	2.6	4.2	1.9	2.9
bio19	13.3	19.5	10.2	10.7
elev	1.9	1.3	2.1	1.3

*Note*: The description of environmental variables is shown in Table 1.

**FIGURE 2:**
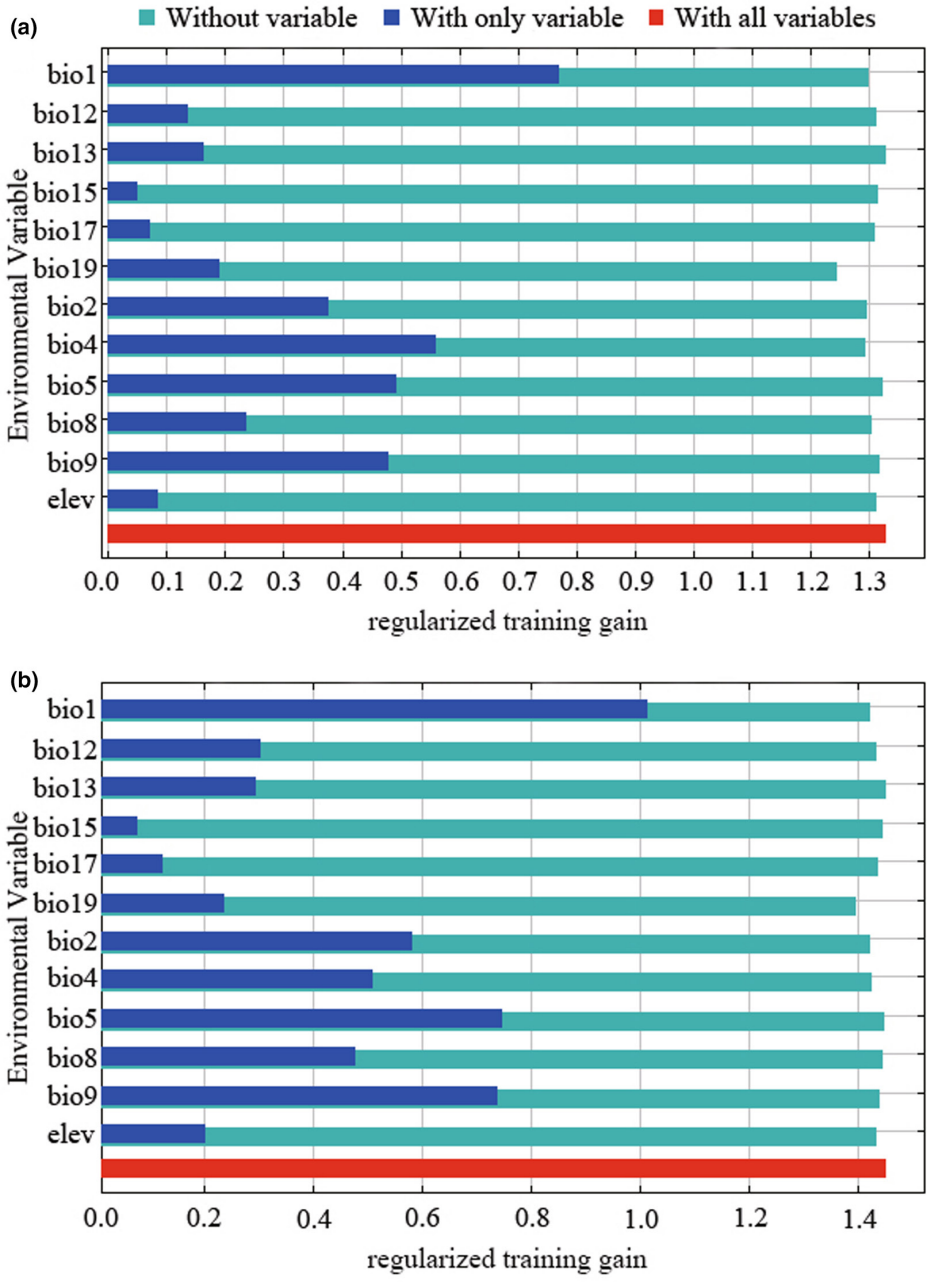
Jackknife of regularized training gain for *Amaranthus palmeri*. The description of environmental variables is shown in Table 1. (a) is the result of WorldClim 1.4; (b) is the result of WorldClim 2.1.

As shown in Figure 2, it gives the results of the jackknife test of variable importance. The regularized training gain is used to judge the importance of environmental variables. For WorldClim1.4, the environmental variables with a high gain when used in isolation are the annual mean temperature (bio1), mean diurnal range (bio2), and temperature seasonality (bio4), which therefore appear to have the most useful information by themselves. In addition, the environmental variable that decreases the gain the most when it is omitted is the precipitation of coldest quarter (bio19), which therefore appears to have the most information that is not present in the other variables. Besides, warmest month (bio5) and mean temperature of driest quarter (bio9) also have a high gain when used in isolation. However, the regularized training gain is still at a high level and there is no significant decrease when they are excluded, respectively. These suggest that bio1, bio2, bio4, and bio19 play dominant roles in the growth of *A. palmeri*, while bio5 and bio9 do not.

Similarly, they are also true in WorldClim2.1. However, the regularized training gains in WorldClim 2.1 are generally higher than in WorldClim 1.4. Overall, the regularized training gain of temperature‐related environmental variables is higher than that of rainfall‐related variables, although the results of jackknife tests with variable importance in WorldClim 1.4 and WorldClim 2.1 are different.

In summary, temperature‐related environmental variables have a greater impact on the suitable habitat of *A. palmeri* than rainfall‐related variables, and the dominant environmental variables affecting the suitable habitat of *A. palmeri* include annual mean temperature (bio1), mean diurnal range (bio2), temperature seasonality (bio4), and precipitation of coldest quarter (bio19).

### 3.3 Response to dominant environmental variables

**FIGURE 3:**
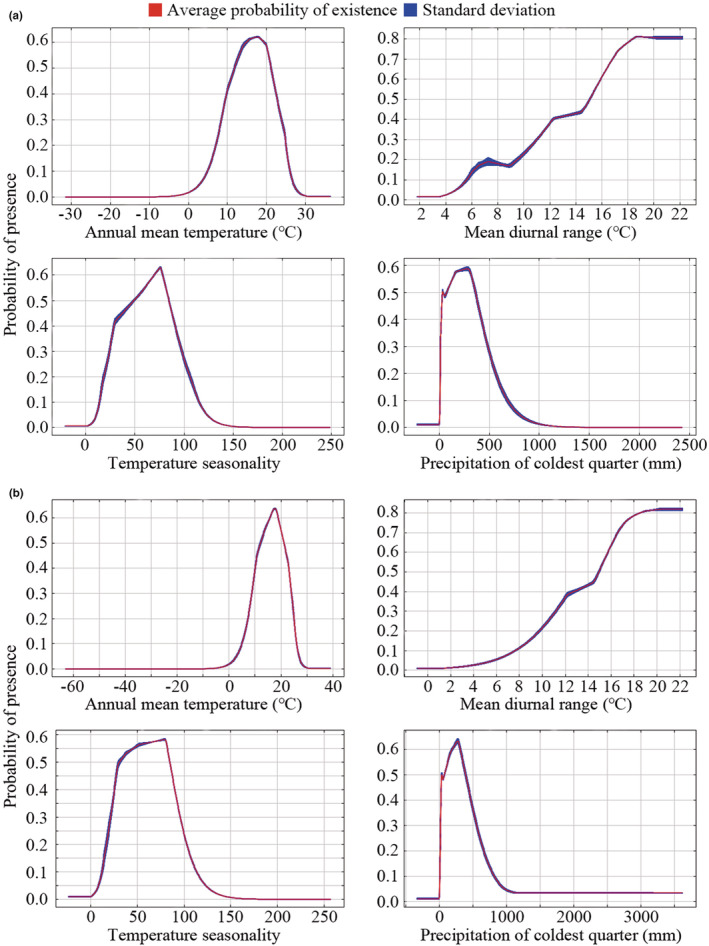
Response of *Amaranthus palmeri* to dominant environmental variables. (a) is the result of WorldClim 1.4; (b) is the result of WorldClim 2.1.

As shown in Figure 3, each curve represents a different model created by using only the corresponding variable. These plots reflect the dependence of predicted suitability on the selected variable and how the predicted probability of presence changes.

For WorldClim1.4, the presence probability of *A. palmeri* shows a typical unimodal response curve to annual mean temperature (bio1). It first increases and then decreases with the increase of bio1. When bio1 is about 18°C, the presence probability reaches a peak. In addition, the presence probability increases with the mean diurnal range (bio2) in the limited data range, and it reaches the maximum and tends to be stable when bio2 is about 20°C. However, the speed of rising is unstable. Besides, the presence probability increases first and then decreases with the increase of temperature seasonality (bio4), and reaches the maximum when bio4 is about 75. Moreover, at the range of precipitation of coldest quarter (bio19) is 0–100 mm, the presence probability fluctuates around 0.5, then increases with the increase of bio19 and reaches the maximum when bio19 is about 250 mm then decreases with the increase of bio19.

For WorldClim2.1, the response of the presence probability of *A. palmeri* to dominant environmental variables is roughly consistent with that in WorldClim 1.4, especially bio1. However, the rising speed of presence probability is more stable with the increase of bio2. In addition, the presence probability increases faster with bio4 when bio4 ranges from 0 to 25, and the peak and the value of bio4 when presence probability reaches the peak are different. Besides, the peak of presence probability is more than 0.6 when bio19 is about 250 mm, and the presence probability eventually decreases to about 0.04 and then tends to be stable.

In summary, the survival probability of *A. palmeri* is close to the unimodal response curve to bio1, bio4, and bio19, indicating that it increases first and then decreases with their increase. In addition, the survival probability is almost a linear response to bio2 in a limited data range, indicating that it is almost proportional to bio2.

### 3.4 Potential distribution under current and future climate scenarios

**FIGURE 4:**
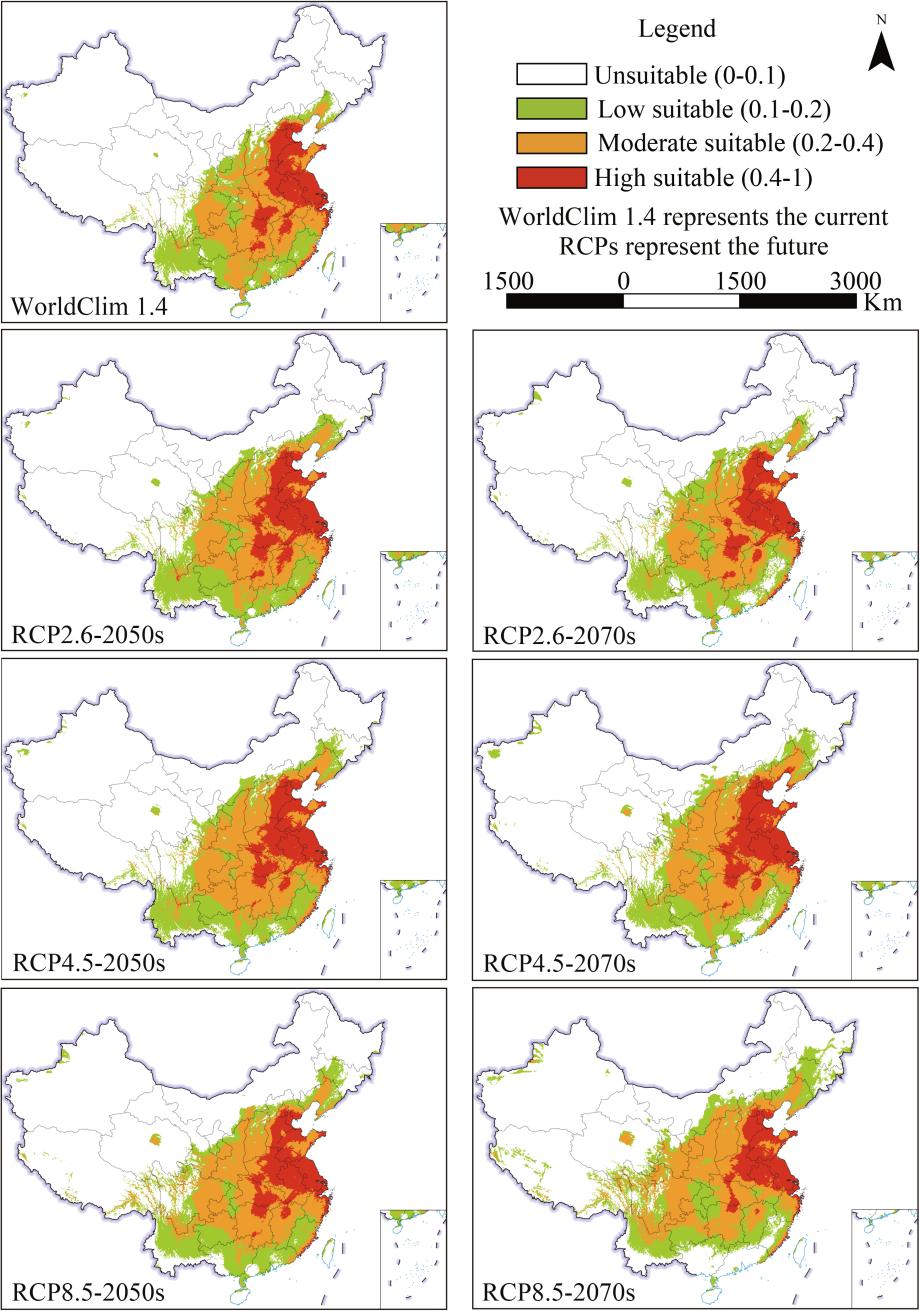
The suitable area of *Amaranthus palmeri* under WorldClim 1.4 and RCPs.

**FIGURE 5:**
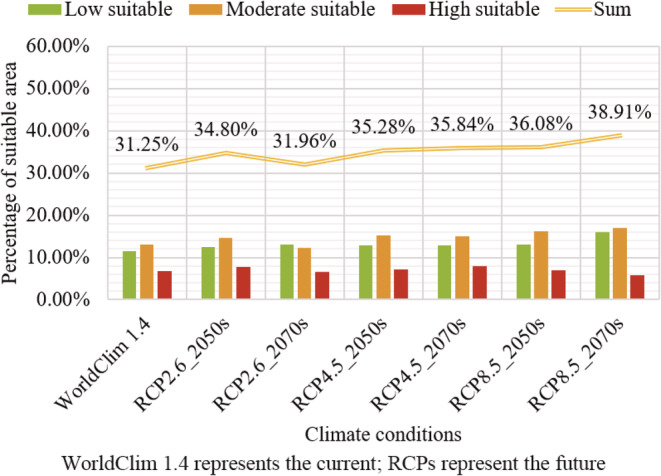
Percentage of suitable areas under WorldClim 1.4 and RCPs.

For WorldClim 1.4 and RCPs, the potential distribution of *A. palmeri* and the area proportion of different suitable habitats are shown in Figures 4 and 5, respectively. At present, the potential suitable area of *A. palmeri* is widely distributed in the central‐east and parts of southwest China. The high suitable areas are focused on the North China Plain. In addition, the high, moderate, low, and unsuitable areas account for 6.80%, 13.05%, 11.39%, and 68.75% of China's total land area, respectively. In the future, the high suitable habitat will be consistent with the current. However, the potential suitable habitat has an obvious expansion trend to the higher latitude and altitude areas. The total suitable area shows an increasing trend. Although the suitable area will decrease under RCP2.6 from the 2050s to 2070s, the suitable area is still slightly larger than the current. In addition, the potential suitable habitat in coastal and border areas in southwest China will disappear to varying degrees, especially in the future 2070s with high carbon emission scenarios (RCP8.5).

**FIGURE 6:**
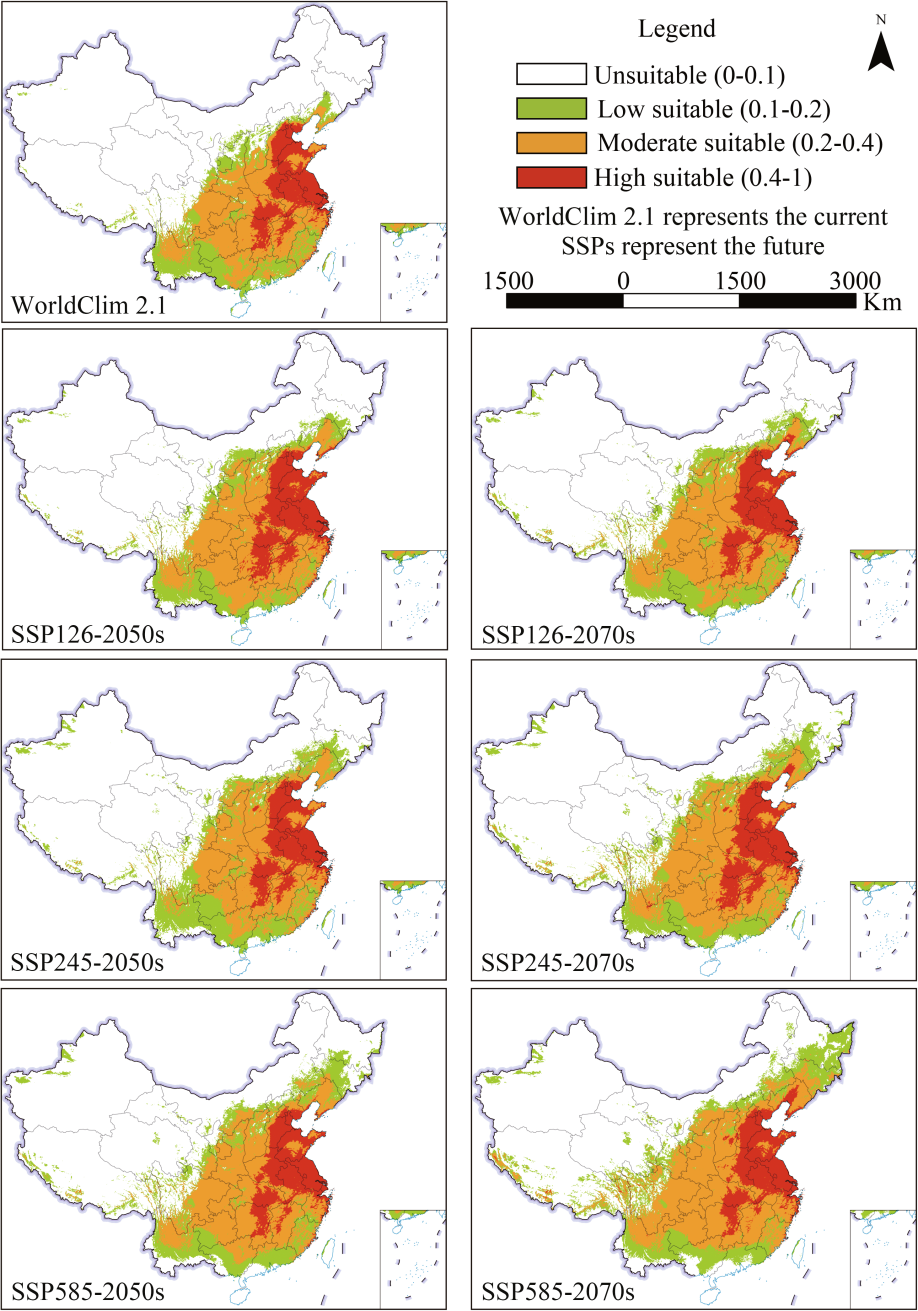
The suitable area of *Amaranthus palmeri* under WorldClim 2.1 and SSPs.

**FIGURE 7:**
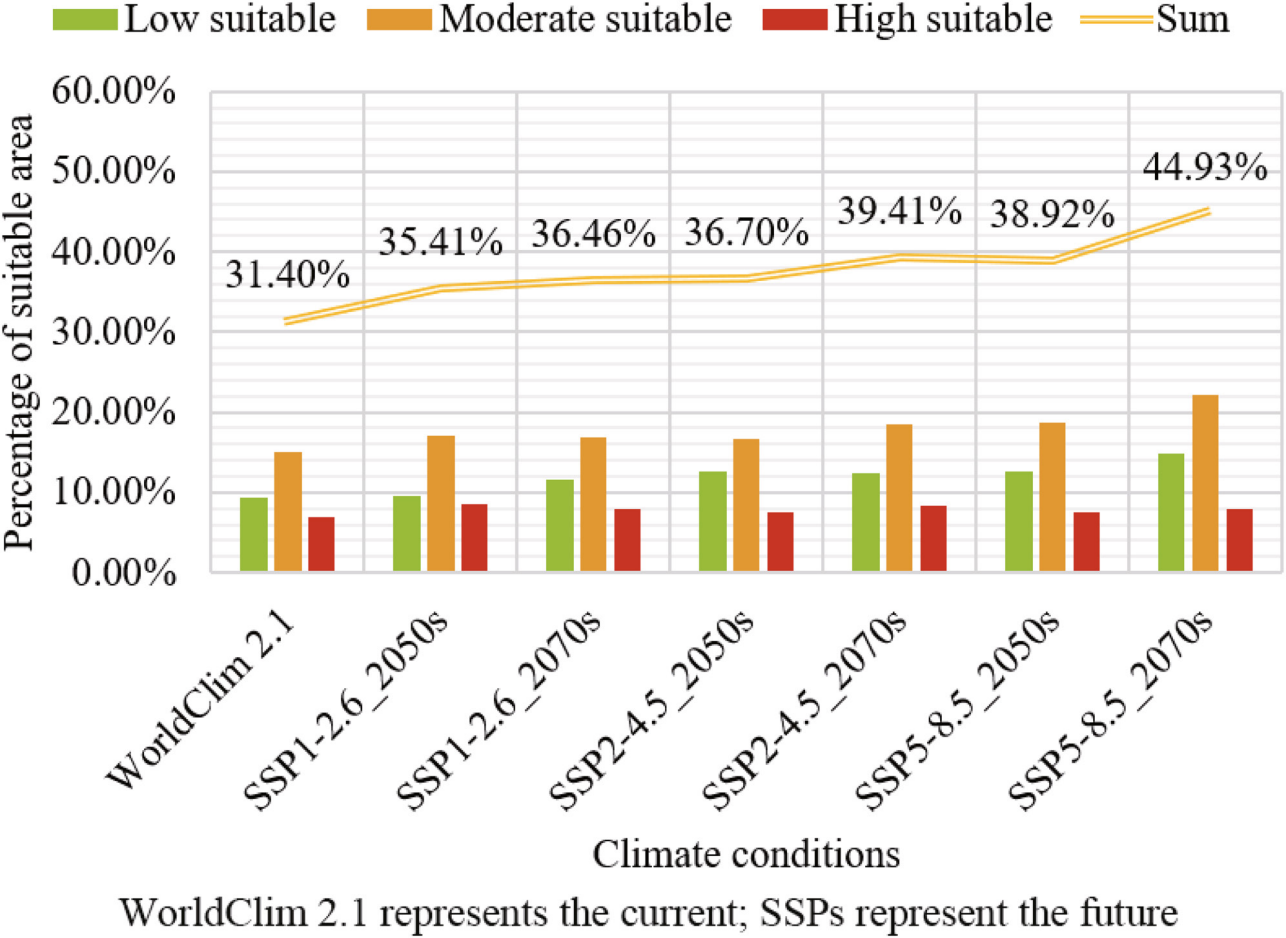
Percentage of suitable areas under WorldClim 2.1 and SSPs.

For WorldClim 2.1 and SSPs, the potential distribution of *A. palmeri* and area proportion of different suitable habitats are shown in Figures 6 and 7, respectively. The potential suitable distribution region of *A. palmeri* is roughly the same as predicted in WorldClim 1.4, but the area proportion of different suitable habitats is different. The high, moderate, low, and unsuitable areas account for 6.98%, 14.96%, 9.46%, and 68.6% of China's total land area, respectively. In the future, the trend of expansion to higher latitudes and altitudes is more significant than that under RCPs. Whether from low to high carbon emissions in the same future period or under the same carbon emission scenario from 2050 to 2070, the total potential suitable area shows an upward trend under SSPs. In addition, the potential suitable habitat in coastal and border areas in southwest China will also disappear to varying degrees, but the loss is smaller than predicted in RCPs.

In summary, the current potential suitable area of *A. palmeri* is widely distributed in the central‐east and parts of southwest China. The high suitable regions are focused on the North China Plain. The total suitable area under WorldClim 1.4 and WorldClim 2.1 is 31.25% and 31.4%, respectively. The suitable area will show an expansion trend in the future, and the expansion under SSPs is greater than that of RCPs.

### 3.5 Change in the suitable area under future climate scenarios

**FIGURE 8:**
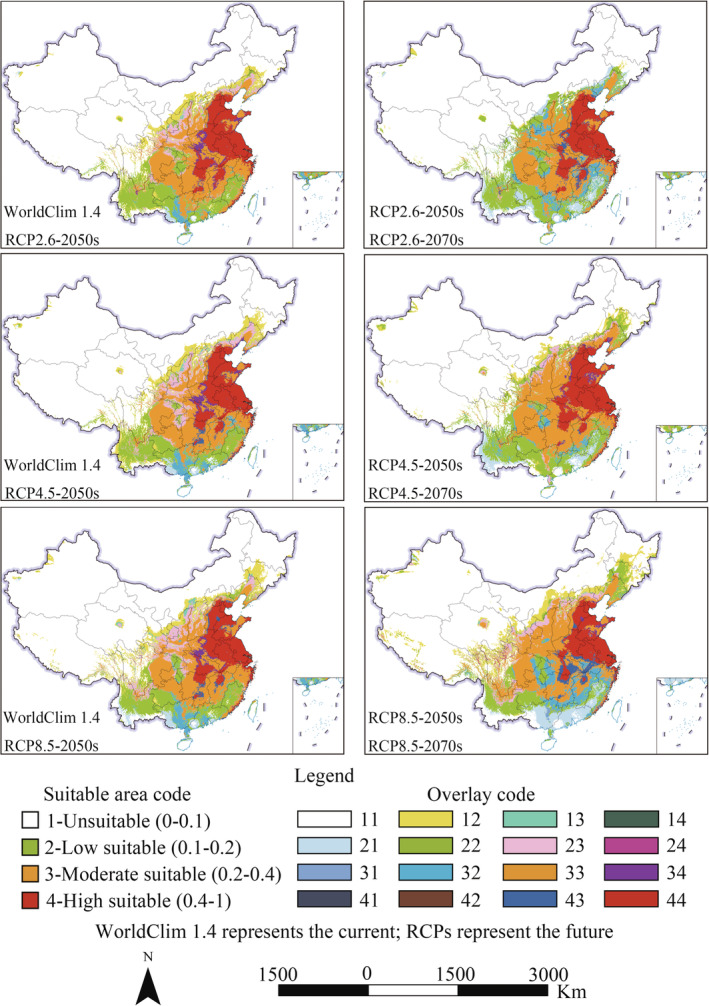
Changes in suitable area of *Amaranthus palmeri* under WorldClim 1.4 and RCPs. The overlay code description is shown in Table 4.

For WorldClim 1.4 and RCPs, the change in the suitable area of *A. palmeri* is shown in Figure 8. From the current to the 2050s under RCPs, the suitable area will show a continuous expansion trend with increased carbon emissions. At the same time, it will show a certain loss of suitable area and a decline in the suitable degree in the southwest coastal and border areas. From the 2050s to the 2070s, the suitable areas will shrink in large areas and barely expand under RCP2.6, the expansion and contraction range are all small under RCP4.5, and the range of expansion and contraction is the largest under RCP8.5.

**FIGURE 9:**
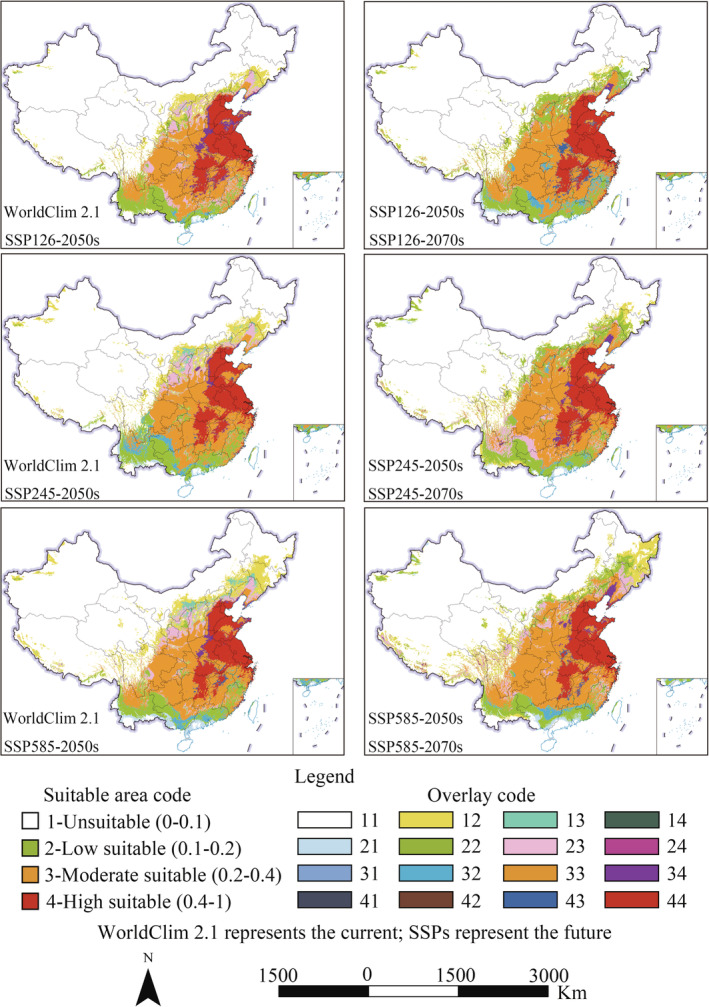
Changes in suitable area of *Amaranthus palmeri* under WorldClim 2.1 and SSPs. The overlay code description is shown in Table 4.

For WorldClim 2.1 and SSPs, the change in the suitable area of *A. palmeri* is shown in Figure 9. From the current to the 2050s and from the 2050s to the 2070s, the range of suitable areas expands more widely with the carbon emissions rise. In addition, the contraction changes little under SSPs.

In summary, the suitable habitats expansion trend of *A. palmeri* will be greater than the contraction, and the contraction trend of RCPs is more obvious than that of SSPs. Overall, suitable habitats will migrate to high latitudes.

## 4 DISCUSSION

### 4.1 Reliability of model predictions

According to the existing distribution information of *A. palmeri*, its distribution in China is unbalanced, and the data record is incomplete. If only the distribution information in China was used to construct the model, it might not be able to capture all the survival potential (Jiménez‐Valverde et al., 2011). Nevertheless, if only the native data is used to construct the model, it is impossible to explain the niche change of the invaded area (Olivier & Antoine, 2008). Li et al. (2015) pointed out that the ecological niche of American, European and Asian populations of *A. palmeri* showed different degrees of overlap. The niche occupied by European and Asian populations showed expansion and vacancy compared with the native populations of the Americas. Hence, we used the global distribution data of *A. palmeri* including the native and invasive areas to obtain the best hypothesis of the potential distribution.

The occurrence degree and sampling factors may cause the data to be concentrated in a geographic space, and redundant data exists while the model runs (Boria et al., 2014). Therefore, our study created the fishnet based on the spatial resolution of the environment layer to filter data. And only one sample point was retained in each grid to ensure no redundant data in the same geographic unit. Additionally, we reduced the dimension of highly correlated environmental variables by multiple collinearity analysis (Jiménez‐Valverde et al., 2011), and selected predictive factors related to the physiological needs of *A. palmeri*. These all improve the accuracy of the model prediction. According to the result, the AUC, Kappa, and TSS values of the models in our study all reached 0.8. The potential suitable areas of the model predicted can completely cover the distribution points of *A. palmeri* in China, indicating that the model prediction results were reliable.

### 4.2 Dominant environmental variables

The key environmental variables related to temperature include annual mean temperature, mean diurnal range, and temperature seasonality. As a C_4_ plant, the appearance and distribution of *A. palmeri* are closely related to the high temperature and arid environment (Ehleringer et al., 1997). *Amaranthus palmeri* is mainly distributed in tropical, subtropical and temperate regions (Xu et al., 2013). Plant growth is closely related to photosynthesis, which usually presents a unimodal response curve to temperature change. Under the condition of sufficient soil moisture, the photosynthesis of *A. palmeri* first increased and then decreased with the leaf temperature. And ninety percent of the peak photosynthetic rate occurred within 36–46°C (the peak leaf temperature was 42°C). The photosynthetic rate decreased relatively fast on both sides of the optimal leaf temperature range (Ehleringer, 1983). In addition, high temperatures can cause secondary dormancy of seeds and reduce the germination rate (Jha et al., 2010; Steckel et al., 2004). According to field observations, most populations of *A. palmeri* occur only vegetative growth in the northeastern temperate region with relatively low annual mean temperature (Cao et al., 2020). Therefore, the survival potential of *A. palmeri* could be limited in areas with relatively high and low annual mean temperatures. The annual mean temperature was also determined as the key factor affecting the suitability of *A. palmeri* in other model prediction research (Kistner & Hatfield, 2018; Runquist et al., 2019). The result of our study is consistent with the above.

Both mean diurnal range and temperature seasonality represent temperature fluctuations. *Amaranthus palmeri* emerged in the whole growing season from spring to autumn. Few emerged in the late growth period, and the plants observed after July were <10% (Piskackova et al., 2021). It has been proved that the germination temperature requirements of *A. palmeri* are different during the 12 months in a year after seed maturation, and the germination ability is significantly affected by temperature seasonally (Jha et al., 2010). All of these indicate that *A. palmeri* is affected by seasonal temperature fluctuations. According to previous studies, the germination rate of *A. palmeri* seeds increased under alternating temperatures compared with constant temperature (Ji et al., 2021; Steckel et al., 2004). When the alternating temperature is at 5°C, the germination rate is only 8%, and with the increase of alternating temperatures, the germination rate is also increasing (Steckel et al., 2004). In another study, the germination rate reached 99.7% at 35°C/15°C in half a month (Ji et al., 2021). In our study, the survival probability of *A. palmeri* showed a linear response in the limited range of mean diurnal range, which was consistent with the results of previous studies. The changes of survival probability with temperature fluctuation further proved that the annual mean temperature played an important role in the survival potential of *A. palmeri*.

Only precipitation of coldest quarter is the important environmental variable associated with rainfall. Rainfall in a particular season seems to be an important factor in plant invasion (Bradley et al., 2009). *Amaranthus palmeri* is an opportunist, which can germinate rapidly in response to water availability in arid environments (Ehleringer, 1983). Studies have shown that *A. palmeri* increased sharply within 1 month of emergence, indicating a rapid rise under favorable conditions (Piskackova et al., 2021). The precipitation of the coldest quarter directly affects soil moisture accumulation before germination. It can be seen from the response curve that when the precipitation of coldest quarter was relatively low, the survival probability showed a rapid increase trend, which indicated the rapid response to water. In addition, the growth, reproduction, and seed germination of *A. palmeri* under no water stress were better than those under water stress (Chahal et al., 2018), which further indicated that the survival probability increased under sufficient water. However, the seed germination rate was significantly reduced under severe flooding conditions, and the presence of fungi resulted in seed rot and decreased seed vigor (Franca et al., 2020). The result in our study was consistent with the previous research results. It is worth noting that the survival probability is relatively high within 500 mm of precipitation of coldest quarter, and the coldest‐season rainfall in China is within this range except in Taiwan Province. According to literature and reports, *A. palmeri* can grow in a variety of habitats with large differences in soil moisture such as farmland, roadside, riverside, and desert (Li, 2003). These indicate that rainfall has little contribution to distribution, but has some influence on growth. Hence, it may be that the relative resistance to waterlogging and drought of *A. palmeri* leads to the contribution of rainfall factors.

### 4.3 Potential distribution and migration

The joint action of different dominant environments restricts the potential distribution of *A. palmeri* in China. The potential distribution area mainly belongs to subtropical and temperate monsoon climate with an annual mean temperature at 8–22°C and has a certain temperature fluctuation. Although distributed in tropical monsoon climate regions, the distribution area is small and has a low survival probability. Because the appearance of *A. palmeri* is proportional to temperature and inversely proportional to rainfall within a certain range (Ehleringer et al., 1997), these areas have frequent rainfall, small temperature fluctuations, and high temperatures throughout the year with an annual mean temperature above 22°C.

The prediction results of SDMs vary with occurrence data, environmental variables, prediction methods and classification methods (Iverson et al., 2017; Zhang et al., 2011). For further comparison with the results of other studies, the coincidence degree comparison was carried out by overlapping the color blocks of different suitable levels in the prediction maps of different research results based on the number of pixels in PS version 2022. When comparing WorldClim 1.4 and WorldClim 2.1 with Li et al. (2015), the coincidence degree of the suitable area reached 98.14% and 97.64%, and the highly suitable area reached 50.16% and 48.54%, respectively. However, the color blocks in the contrast area cannot be accurately selected in extracting color blocks because the color distinction of Li et al. (2015) is not obvious enough. Therefore, there was a certain deviation in the comparison results of the coincidence degree in the high suitable area. Nevertheless, from the prediction results, our study is consistent with the range of suitable areas predicted by Li et al. (2015) and Xu et al. (2013).

The temperature and rainfall changes in coastal and border areas in southwest China will intensify, especially the annual mean temperature will rise. Climate change will further restrict the occurrence of *A. palmeri* in these areas, leading to the loss of suitable habitats in cases of low suitability. In addition, global warming in the future will increase in accumulated temperature, thereby extending the growth period of *A. palmeri* in high latitudes. And the climatic suitability in the northern hemisphere will improve (Kistner & Hatfield, 2018). In this study, the expansion of *A. palmeri* to high latitude is consistent with the above. The correlation between phenotypic variation and temperature in different latitude populations of *A. palmeri* shows strong climate adaptability (Cao et al., 2020). The combined effects of climate change and high plasticity will contribute to the invasion and have a broader niche (Cao et al., 2020; Korres & Norsworthy, 2017).

Furthermore, global warming leads to changes in limiting factors for plant invasion in the mountain ecosystem, such as increased plant growth seasons by increasing temperature, reduced frequency and duration of frost and snow, and so on (Chen et al., 2011). These may weaken the abiotic resistance of most mountain ecosystems to plant invasion. There are signs that alien species are expanding upward, and more than 1000 alien species have been naturalized in global high‐altitude ecosystems (Pauchard et al., 2009). These indicate that climate changes will promote the invasion of alien plants to high‐altitude areas in the future. In our study, *A. palmeri* will expand to high‐altitude areas, especially in high carbon emission scenarios.

Invasive plants are usually more adaptable to the new environment than local plants (Bradley et al., 2009). The increased carbon dioxide concentration in the atmosphere has a direct “fertilization” effect on plants, and changes in temperature and precipitation can also create new environments (Bradley et al., 2009). These will benefit the invasive plants. On the other hand, reduced photosynthetic rates and energy limits at lower temperatures may limit the potential range of *A. palmeri*, or at least affect its competitiveness with other plants (Ward et al., 2013). Among our predictions under future climate scenarios, the lowest temperature rise is under RCP2.6 (Shen & Wang, 2013), limiting the global average temperature rise to 2°C by 2100. In our study, the increase of suitable area under RCPs is less than that under SSPs, and the minimum increase under RCP2.6. Therefore, we consider that low‐carbon emissions may be an important factor restricting the suitable habitat. Compared with the SSPs, the contraction trend of RCPs is more obvious. This may be because the future climate scenarios SSPs consider socio‐economic development factors so that SSPs have substantially higher CO_2_ emissions than RCPs, with correspondingly larger cuts in non‐CO_2_ emissions. Therefore, the prediction of the potential distribution of *A. palmeri* under SSPs is more stable than that under RCPs. However, the important environmental variables that cause the migration remain to be further studied.

### 4.4 Research significance and prospect

Climate change and biological invasion have directly affected biodiversity and food security. Many observations show that climate change significantly impacts biodiversity by affecting the frequency and extent of pests and diseases, the phenology, biotic interactions, distribution, and abundance of species (Habibullah et al., 2022; Wu et al., 2009). Climate change and extreme weather events, such as low or high temperature stress, changes in rainfall patterns, rainstorms, and droughts, not only threaten crop productivity through biological structures such as affecting crop cycles, pest invasions, but also cause difficulties in agricultural activities (Farooq et al., 2022; Zhou et al., 2013). In addition, the increased carbon dioxide promotes weed growth and reduces herbicide efficacy, leading to increased competition between weeds and crops (Ziska et al., 2004, 2011). The confluence of climate change and biological invasion may profoundly impact biodiversity and agricultural production.

Physiological plasticity, varied tolerance ranges, phenotypic plasticity, and high fecundity and transmission capacity may make invasive alien plants easy to cope with global change (Bradley et al., 2009; Qin et al., 2018). However, types of research prove that some components of global change may promote or hinder species invasion. For example, global warming may increase the speed of diffusion and growth of organisms controlled by temperature (Walther et al., 2009). The photosynthetic rate of *Mikania micrantha*, *Wedelia trilobata*, and *Ipomoea cairica* increased by 67.1% on average when they were exposed to elevated CO_2_ concentrations. And the elevated CO_2_ also led to significant changes in biomass allocation and morphology of *M. micrantha* and *W. trilobata* (Song et al., 2009). Elevated CO_2_ and warming had antagonistic effects on the population growth of *Hypochaeris radicata* and *Leontodon taraxacoides* (Williams et al., 2007). In addition, non‐local plant invasion promotes greenhouse gas emissions, thereby accelerating global climate change (Beyene et al., 2022). Species invasion is related to the synergy of the species characteristics and global change. It is necessary to prepare for the expansion and contraction of invasive species.

*Amaranthus palmeri* was first discovered in China near a railway line dedicated to cooking oil factories in Beijing (Li, 2003). It usually occurs with crop growth, so it is likely to mix with crop harvest and spread with grain transportation. According to the statistical analysis of port interception in China, the seeds of *A. palmeri* were most frequently intercepted from soybean goods originating in the United States and Brazil (Yang et al., 2015). Therefore, *A. palmeri* may be imported into China with imported oil soybeans and other agricultural products. In recent years, *A. palmeri* has been colonized in many provinces across low‐latitude tropical regions and high‐latitude temperate regions in China, especially in the Beijing‐Tianjin‐Hebei region. Other areas with distribution have relatively few records. According to the distribution of *A. palmeri* in China and the diffusion models in the distribution area of invasive species proposed by Shigesada and Kawasaki (1997), it can be judged that *A. palmeri* is spreading in both short and long distances. Nowadays, the increasing frequency of human activities and the rapid development of transportation and logistics greatly increase the risk of species spreading along specific vectors such as transportation (Horvitz et al., 2017). However, it is necessary to verify further the correlation between the distribution of *A. palmeri* and the road to determine whether a road is the main way of its long‐distance dispersal.

On the one hand, as a C_4_ plant, *A. palmeri* has the characteristics of high carbon dioxide utilization and low water requirement for growth, which has certain advantages in dealing with the increase of atmospheric carbon dioxide and more frequent and severe drought (Ward et al., 1999). On the other hand, changes in temperature and precipitation can create a new environment (Bradley et al., 2009). Some traits of invasive species will make them adapt to the new environment faster and better than native species (Engel et al., 2011). Studies have shown that *A. palmeri* can adapt to different environmental conditions by adjusting its phenotypic traits or genetic variations (Cao et al., 2020; Korres & Norsworthy, 2017), which plays an important role in its colonization and invasion of new environments under climate change. It has been found that most of *A. palmeri* were vegetative growth in the northeast temperate zone of China with low annual mean temperature (Cao et al., 2020). With the development of global warming, it may improve the climate suitability of *A. palmeri* in the high latitudes of the northern hemisphere to promote reproductive growth.

The current and future potential suitable areas of *A. palmeri* in China are consistent with the production areas of soybean, corn and other crops. Early germination behavior and rapid growth characteristics can promote biomass accumulation and lead to the successful establishment of *A. palmeri* in the agricultural field (Ehleringer, 1983; Piskackova et al., 2021; Steckel et al., 2004). And it cannot be effectively controlled for a long time due to the evolved herbicide resistance of *A. palmeri* (Ward et al., 2013). Although *A. palmeri* has been recorded in many provinces of China, its invasion potential has not been fully exploited. Early monitoring and warning and active rapid responses are important means to prevent the introduction from foreign countries and further spread in China. In this study, the dominant environmental variables affecting the suitability habitat of *A. palmeri* were determined and the potentially suitable areas in China under current and future climate scenarios were predicted, which is beneficial to the accurate policy and effective management of the continuous invasion and future spread.

However, our study still has many deficiencies. Firstly, species distribution is subject to interactions between biological and abiotic factors (Boivin et al., 2016; Gama et al., 2015; Lewis et al., 2017). Our study mainly considered the influence of climate on the distribution of *A. palmeri*. The invasion potential of species, the interaction between organisms, soil conditions, and land use play an important role in the distribution of invasive plants (Hong et al., 2021; Manzoor et al., 2021; Potter & Bowman, 2020). In addition, transportation construction, trade activities, and water conservancy projects are extremely important for spreading invasive species (Adhikari et al., 2021; Horvitz et al., 2017; Liu et al., 2017). Therefore, a realistic risk assessment must integrate ecology, geography, climate science and economic development (Bradley et al., 2009). Secondly, the prediction results of models will be affected due to different global climate models and carbon emission scenarios (Jiang et al., 2022). Different regions have different carbon emission conditions and emission reduction policies, so the prediction of species distribution under future climate change should be combined with the actual emission targets and trends (Gütschow et al., 2021). Thirdly, the model itself is an uncertain factor, and there is no general standard for algorithm selection when modeling the geographical distribution of species (Silva et al., 2021; Zhao, Cui, et al., 2021). Different models have different preferences due to the different operating principles. Biomod2 package enables weighted integration of multiple models. Thus, it is increasingly used to predict the potential distribution of species (Fang et al., 2020; Pacifici et al., 2019). In further research, it is necessary to carry out a field survey of risk areas in time and use updated data combined with more variables to conduct further regional scale analysis based on the ensemble model. And combined with China's carbon emissions policy, make it more in line with China's actual situation forecast.

## 5 CONCLUSION

In this study, the MaxEnt model was used to analyze the dominant environmental variables affecting the suitability habitat of *A. palmeri* and predict its potential distribution in China under current and future climate scenarios. Our research shows that temperature‐related environmental variables have a greater impact on the suitable habitat of *A. palmeri* than rainfall‐related variables. At present, the potential distribution of *A. palmeri* is widely distributed in the central‐east and parts of southwest China. The highly suitable areas are focused on the North China Plain. In the future, the suitable habitat will expand significantly to higher latitudes and altitudes in response to global warming. Therefore, it is necessary to establish early monitoring and warning, rapid response, and real‐time control systems to prevent further spread and outbreaks.

## AUTHOR CONTRIBUTIONS

**Xinyi Zhang:** Data curation (lead); formal analysis (lead); methodology (lead); visualization (lead); writing – original draft (lead); writing – review and editing (equal). **Jian Zhao:** Conceptualization (lead); funding acquisition (lead); project administration (lead); supervision (supporting); writing – review and editing (equal). **Miaomiao Wang:** Supervision (supporting); writing – review and editing (equal). **Zhipeng Li:** Funding acquisition (equal); project administration (equal); supervision (supporting); writing – review and editing (supporting). **Sheng Lin:** Supervision (supporting); writing – review and editing (supporting). **Hong Chen:** Supervision (supporting); writing – review and editing (supporting).

## CONFLICT OF INTEREST

The authors declare that they have no conflict of interest.

## Data Availability

Datasets used in this study are available online in Dryad at https://doi.org/10.5061/dryad.zpc866tc4.
